# Finger shortening for Dupuytren’s disease-induced severe PIP joint flexion contracture of the little finger: A report of two cases

**DOI:** 10.1051/sicotj/2021005

**Published:** 2021-03-08

**Authors:** Yoko Ito, Kiyohito Naito, Nana Nagura, Yoichi Sugiyama, Hiroyuki Obata, Ayaka Kaneko, Kenji Goto, Kazuo Kaneko, Muneaki Ishijima

**Affiliations:** Department of Orthopaedics, Juntendo University Faculty of Medicine 2-1-1 Hongo Bunkyo-ku 113-8421 Tokyo Japan

**Keywords:** Finger shortening, Dupuytren’s disease, Severe PIP joint flexion contracture, Little finger

## Abstract

When severe proximal interphalangeal (PIP) joint flexion contracture is induced in the little finger by Dupuytren’s disease, it interferes with activities of daily living. To extend the little finger, open fasciectomy is selected as a general treatment method. However, postoperative complications have been frequently reported. To solve these problems, finger shortening was undertaken. In this study, we treated two cases of Dupuytren’s disease manifesting severe PIP joint flexion contracture of the little finger with finger shortening by proximodistal interphalangeal (PDIP) fusion in which the middle phalanx is resected and the residual distal and proximal phalanges are fused. For flexion contracture of the MP joint, a percutaneous aponeurotomy using an 18G needle was performed to obtain the extended position of the MP joint. Favorable outcomes with high patient satisfaction, including esthetic aspects of retaining the finger with the nail without complication, were achieved. We report this challenging treatment and its discussion.

## Introduction

When severe proximal interphalangeal (PIP) joint flexion contracture is induced in the little finger by Dupuytren’s disease, it interferes with activities of daily living, such as face washing, causes skin disorders, such as tinea manus, and has harmful esthetic effects [1]. To extend the little finger, open fasciectomy is selected as a general treatment method even though severe PIP joint flexion contracture is observed [2–4]. However, postoperative skin necrosis and infection, and recurrence have been frequently reported. Moreover, the requirement of skin grafting and flap surgery in cases with difficulty in covering the wound has been occasionally reported [5, 6]. Furthermore, there are intractable cases with repeated recurrence inevitably treated by amputation after multiple surgeries even though finger blood flow and nails were normal to be reported [1, 7]. Tonkin et al. reported that amputation was inevitably selected in approximately 9% of recurrent cases in the little finger [8]. However, amputation is not necessarily a satisfactory surgical procedure and it has risks of flexion deformity of the finger, amputation neuroma-induced pain, and Phantom pain syndrome [9], i.e., there are many problems to be improved in the treatment of Dupuytren’s disease of the little finger.

To solve these problems, Watson and Lovallo designed finger shortening by bone resection of the proximal phalanx [10], and Honecker et al. proposed proximodistal interphalangeal (PDIP) fusion in which the middle phalanx is resected and the residual distal and proximal phalanges are fused [11]. In this study, we treated two cases of Dupuytren’s disease manifesting severe PIP joint flexion contracture of the little finger with finger shortening by PDIP fusion. We report this challenging treatment and its discussion.

## Case report

### Patients

The study was carried out in accordance with the Declaration of Helsinki and the appropriate ethical framework. Written informed consent was obtained from the patients for publication of this case report and any accompanying images.

The subjects were two patients who underwent finger shortening for severe PIP joint flexion contracture of the little finger caused by Dupuytren’s disease between January 2012 and December 2017 at our hospital. The first case was a 59-year-old male with no notable past medical history. The disease was Dupuytren’s disease manifesting severe PIP joint flexion contracture (flexion 100°) and metacarpophalangeal (MP) joint flexion contracture (flexion 45°) in the left little finger. There was no deformity or osteoarthritis on the distal interphalangeal (DIP) joint. The range of motion of the DIP joint was flexion 45° and extension 0°. The preoperative Tubiana classification was stage 4, grip strength was 32 kg, visual analogue scale (VAS) was 0/10, and quick disabilities of the arm, shoulder, and hand (Q-DASH) score was 25.00/100. The patient requested esthetic acquisition of the finger extension position. The second patient was an 81-year-old male with no notable past medical history. The disease was Dupuytren’s disease manifesting severe PIP joint flexion contracture (flexion 90°) and MP joint flexion contracture (flexion 30°) in the left little finger. There was no deformity or osteoarthritis on the distal interphalangeal (DIP) joint. The range of motion of the DIP joint was flexion 25° and extension 0°. The preoperative Tubiana classification was stage 3, grip strength was 16 kg, VAS was 3/10, and Q-DASH score was 36.36/100. The patient requested surgery because the little finger was an obstacle in washing his face and interfered with activities of daily living.

### Surgical technique

**Figure 1:**
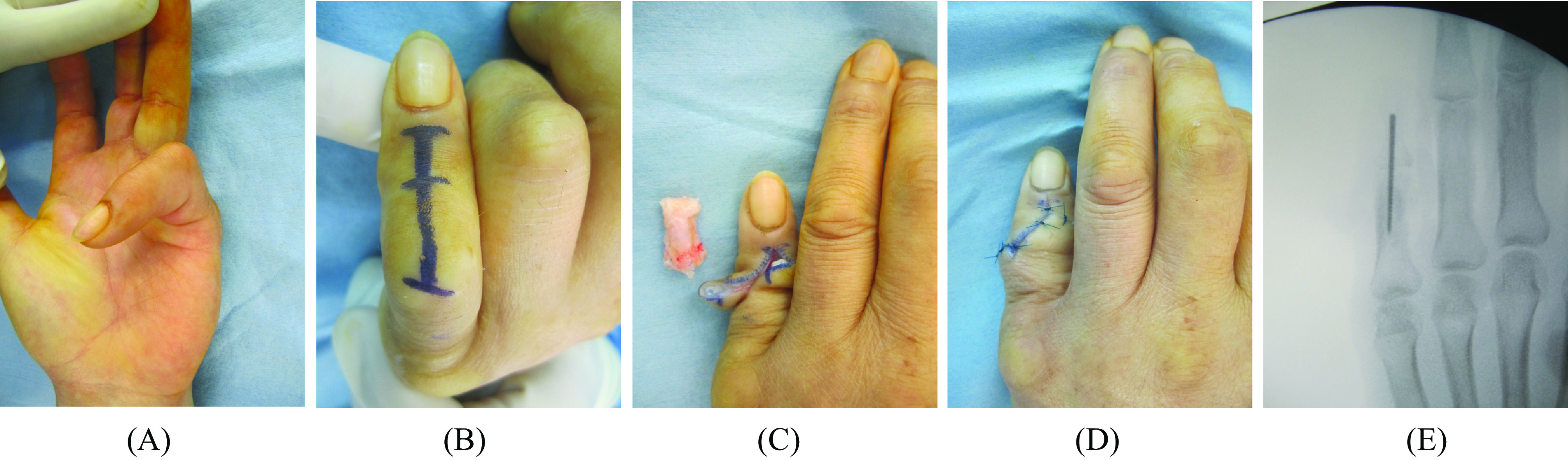
Intraoperative findings. (A) Dupuytren’s disease-induced severe PIP joint flexion contracture of the little finger was observed. (B) A dorsal approach was designed to resect the middle phalanx. (C) The extension position of the little finger was acquired by resection of the middle phalanx. (D and E) After resection of the middle phalanx, articular cartilages distal to the proximal phalanx and proximal to the distal phalanx were removed, and these 2 bones were fixed at the little finger extension position using Break-Away Screw (Acutwist^®^, Nihon Medical Next, Osaka Japan) ((D) Macroscopic findings of the little finger after surgery, (E) intraoperative fluoroscopic image).

Surgery was performed under the brachial plexus block. First, for flexion contracture of the MP joint, a percutaneous aponeurotomy using an 18G needle was performed to obtain the extended position of the MP joint. Extension 0° in MP joint was obtained in both cases by a percutaneous aponeurotomy. To release severe flexion contracture of the PIP joint (Figure 1A), it was not approached from the palmar side, but from the dorsal side (Figure 1B). The finger extension position was acquired by dissection of soft tissue around the middle phalanx and resection of the middle phalanx (Figure 1C). After resection of the middle phalanx, the articular cartilages distal to the proximal phalanx and proximal to the distal phalanx were removed, and the two bones were fixed using Break-Away Screw (Acutwist^®^, Nihon Medical Next, Osaka, Japan) (Figures 1D and 1E). PIPD joint was able to be fixed at extension 0° in both patients. The operative time was 60 min in the first case and 45 min in the second case. At three weeks after surgery, splint fixation was applied to fix the region distal to the proximal phalanx at the extension position.

### Outcomes

**Figure 2:**
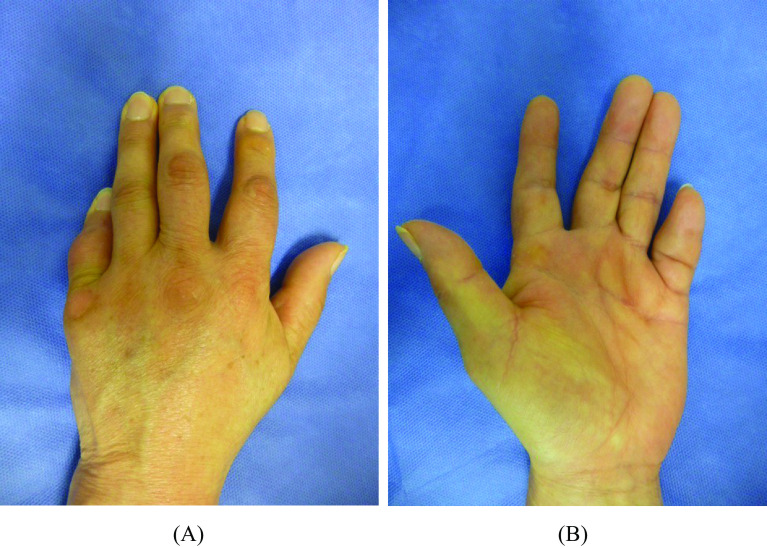
Clinical findings on the final follow-up (2 years after surgery) in Case 1. (A and B) The little finger extension position set by surgery was acquired without recurrence and the surgery was esthetically highly satisfactory without nail deformation ((A) dorsal side, (B) palmar side).

**Figure 3:**
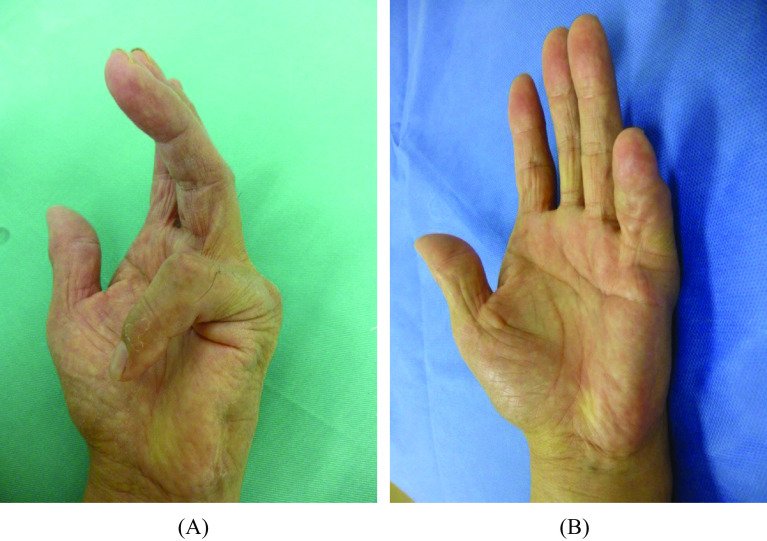
Clinical findings in Case 2. (A) Dupuytren’s disease-induced severe PIP joint flexion contracture of the little finger was observed before surgery. (B) The little finger extension position set by surgery was acquired without recurrence and the surgery was esthetically highly satisfactory on the final follow-up (2 years after surgery).

**Figure 4:**
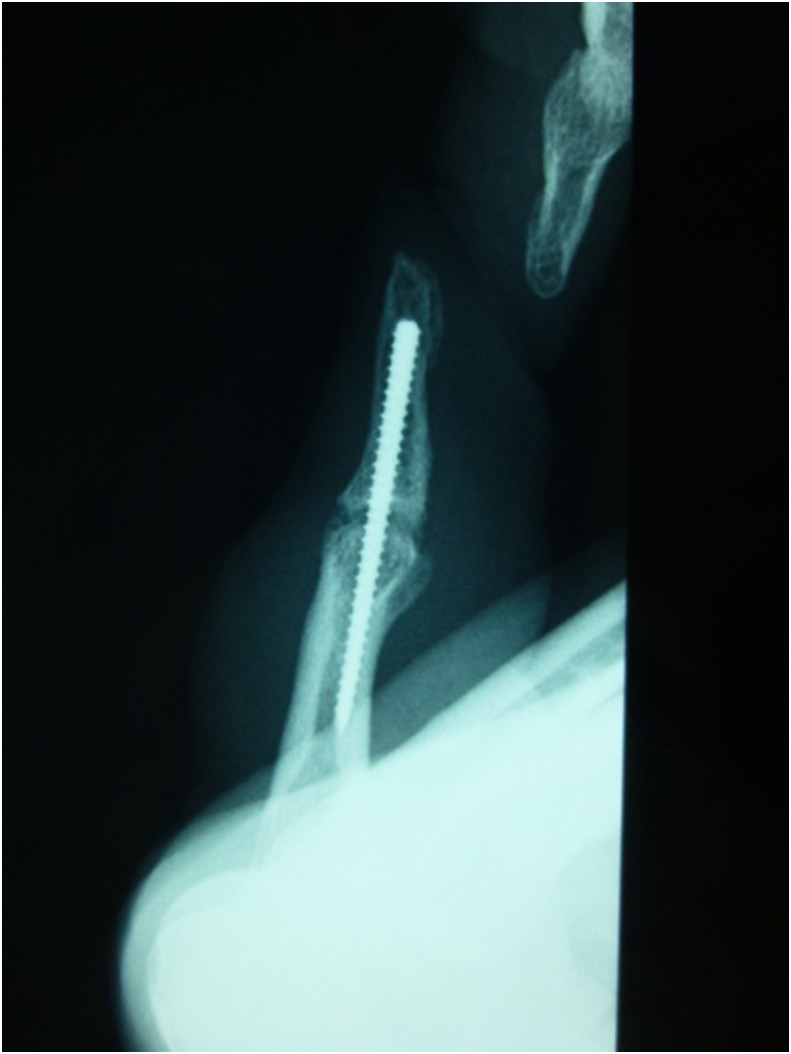
Plain radiography on the final follow-up (2 years after surgery). Bone union of the distal and proximal phalanges was confirmed on plain radiography.

The patients were clinically evaluated two years after surgery. In the first case, the extension position of the little finger was acquired, VAS was 0/10, the Q-DASH score was 0/100, and grip strength was 21 kg (65% of that before surgery) (Figures 2A and 2B). The extension position of the little finger was also acquired in the second case. The VAS was 2/10, the Q-DASH score was 6.82/100, and the grip strength was 11 kg (68% of that before surgery) (Figures 3A and 3B). Bone union at extension 0° in PIPD joint was acquired in both patients (Figure 4). The extension of 0° in the MP joint which was obtained by a percutaneous aponeurotomy did not recur until final follow-up in both cases.

## Discussion

In surgically treated recurrent cases, the skin condition was worse than that after the first surgery, and the incidence of complications, such as skin necrosis and infection, increased [6, 12]. In Dupuytren’s disease manifesting in the little finger, recurrent cases of severe contractures of the PIP joint have been occasionally reported and some patients requested amputation [13]. Finger shortening was designed to solve these problems [10, 11]. The advantages of this treatment are the simplicity of the surgical procedure, the safety of the dorsal approach without the volar approach, the applicability to patients after multiple surgeries, the aesthetic advantage over amputation, and the high patient satisfaction [10, 11].

Of course, there are some disadvantages to finger shortening. First, from a cosmetic perspective, there are concerns about the acceptance of female patients. Raimbeau et al. performed this procedure on seven female patients and all of whom were cosmetically satisfied [12]. Furthermore, as the fingertip with nail remains, patient satisfaction with the esthetic aspect of this surgical procedure is high compared with that of amputation [11, 12, 14]. Second, finger shortening has the disadvantage of finger flexion, as the distance between the finger pulp and palm can be approximately 1 cm [13]. In addition, the inability to move the phalangeal joint has been reported as a disadvantage of this surgical procedure [11]. However, the shortened finger, which is stabilized the extended position by finger shortening, allows the patient to grasp an object (limited to about 7.5–10 cm objects) [12]. Moreover, activities of daily living, such as wearing gloves and shaking hands, are greatly improved by eliminating hook-shaped fingers [12]. Eliminating hook-shaped fingers makes it easier to hold things in daily life [15]. Finally, there are no reports on long-term outcomes, so careful long-term follow-up should be required for recurrence [10, 11]. Not surprisingly, we have given sufficient informed consent to these disadvantages in the present two cases of severe PIP joint flexion contracture prior to surgery. However, the highest priority for these patients was “acquisition of finger extension distal to the MP joint” and these cases were considered good indications for finger shortening.

Although we performed surgery following the procedure reported by Honecker et al. (PDIP fusion) [11], the procedure reported by Watson et al. has an advantage that autonomic movement of the DIP joint is preserved [10]. Both Watson’s procedure and PDIP fusion can reduce the risk of developing neuroma due to the dorsal approach [10, 11, 16]. In addition, it is thought that the recurrence rate will decrease due to the arthrodesis, and past reports also have stated so [12]. However, as the surgical procedure reported by Watson et al. preserves the DIP joint, when Dupuytren’s disease recurs in soft tissue (central cord, lateral cord, and retrovascular cord) distal to the MP joint, the risk of recurrence of the finger flexion position may remain [10]. Moreover, Black and Blazar suggested that the lateral cord and retrovascular cord are responsible for DIP joint contracture [17]. Therefore, we selected the surgical procedure reported by Honecker et al. and tried to reduce the risk of recurrence of the flexion position by making the phalanx a “bone” [11]. At the time of surgery, there was no flexion contracture of the DIP joint in two cases of this study, but it is not possible to determine the origin of a pathological cord [18]. At first glance, the fusion of a non-pathological DIP joint looks disappointing, but the presence of lateral cord and retrovascular cord that contribute to DIP joint contracture cannot be ruled out [17, 19]. Therefore, we believe that our procedure in this study is better compared to Watson’s procedure, considering avoiding the recurrence of Dupuytren’s disease.

## Conclusion

Based on this report of two cases, finger shortening by PDIP fusion for Dupuytren’s disease manifesting severe PIP joint flexion contracture of the little finger may be a useful surgical method. The indication of this surgical procedure should be carefully considered in each case. However, finger shortening is capable of overcoming the problems of other surgical procedures in cases in which amputation is considered or multiple surgeries were performed.

## Conflict of interest

The authors declare that they have no conflict of interest.

## References

[1] Blazar PE, Garon MT (2015) Ray resections of the fingers: Indications, techniques, and outcomes. J Am Acad Orthop Surg 23, 476–484. 26209144 10.5435/JAAOS-D-14-00056

[2] Henry M (2014) Dupuytren’s disease: Current state of the art. Hand (N Y) 9, 1–8. 24570630 10.1007/s11552-013-9563-0 PMC3928378

[3] Rodrigues JN, Becker GW, Ball C, Zhang W, Giele H, Hobby J, Pratt AL, Davis T (2015) Surgery for Dupuytren’s contracture of the fingers. Cochrane Database Syst Rev 2015, CD010143. 10.1002/14651858.CD010143.pub2 PMC6464957 26648251

[4] Vesper US, Mehling IM, Arsalan-Werner A, Sauerbier M (2017) Primary intervention in Dupuytren’s disease. Orthopade 46, 336–341. 28243691 10.1007/s00132-017-3395-5

[5] Eberlin KR, Mudgal CS (2018) Complications of treatment for Dupuytren disease. Hand Clin 34, 387–394. 30012298 10.1016/j.hcl.2018.03.007

[6] van Rijssen AL, Gerbrandy FSJ, Ter Linden H, Klip H, Werker PMN (2006) A comparison of the direct outcomes of percutaneous needle fasciotomy and limited fasciectomy for Dupuytren’s disease: A 6-week follow-up study. J Hand Surg Am 31, 717–725. 16713831 10.1016/j.jhsa.2006.02.021

[7] Degreef I, De Smet L (2009) Dupuytren’s disease: A predominant reason for elective finger amputation in adults. Acta Chir Belg 109, 494–497. 19803262 10.1080/00015458.2009.11680467

[8] Tonkin MA, Burke FD, Varian JP (1985) The proximal interphalangeal joint in Dupuytren’s disease. J Hand Surg Br 10, 358–364. 4078465 10.1016/s0266-7681(85)80062-0

[9] Jensen CM, Haugegaard M, Rasmussen SW (1993) Amputations in the treatment of Dupuytren’s disease. J Hand Surg Br 18, 781–782. 8308443 10.1016/0266-7681(93)90245-b

[10] Watson HK, Lovallo JL (1987) Salvage of severe recurrent Dupuytren’s contracture of the ring and small fingers. J Hand Surg Am 12, 287–289. 3559089 10.1016/s0363-5023(87)80291-5

[11] Honecker S, Hidalgo Diaz JJ, Naito K, Pire E, Prunières G, Facca S, Liverneaux P (2016) Proximodistal interphalangeal arthrodesis of the little finger: A series of 7 cases. Hand Surg Rehabil 35, 262–265. 27781989 10.1016/j.hansur.2016.06.003

[12] Raimbeau G, Bigorre N, Balti W, Rabarin F, Jeudy J, Fouque PA, Cesari B, Saint-Cast Y (2019) Middle phalangectomy with shortening fusion of the fifth finger in Dupyutren’s digital hooks. Hand Surg Rehabil 38, 108–113. 30665870 10.1016/j.hansur.2018.12.003

[13] Eiriksdottir A, Atroshi I (2019) A new finger-preserving procedure as an alternative to amputation in recurrent severe Dupuytren contracture of the small finger. BMC Musculoskelet Disord 20, 323. 31288790 10.1186/s12891-019-2701-2 PMC6617564

[14] Cheng G, Fang G, Hou S, Pan D, Yuan G, Wang Z, Zhang Y, Ding X, Tang H, Yang Z (2007) Aesthetic reconstruction of thumb or finger partial defect with trimmed toe-flap transfer. Microsurgery 27, 74–83. 17295257 10.1002/micr.20310

[15] Mella JR, Guo L, Hung V (2018) Dupuytren’s contracture: An evidence based review. Ann Plast Surg 81, S97–S101. 30161050 10.1097/SAP.0000000000001607

[16] von Campe A, Mende K, Omaren H, Meuli-Simmen C (2012) Painful nodules and cords in Dupuytren disease. J Hand Surg Am 37, 1313–1318. 22560560 10.1016/j.jhsa.2012.03.014

[17] Black EM, Blazar PE (2011) Dupuytren disease: An evolving understanding of an age-old disease. J Am Acad Orthop Surg 19, 746–757. 22134207 10.5435/00124635-201112000-00005

[18] McFarlane RM (1974) Patterns of the diseased fascia in the fingers in Dupuytren’s contracture: Displacement of the neurovascular bundle. Plast Reconstr Surg 54, 31–44. 4832466 10.1097/00006534-197407000-00004

[19] Thoma A, Karpinski M (2017) Involvement of the interosseous and lumbrical muscle-tendon units in the lateral and spiral cords in Dupuytren’s disease of the middle fingers. Plast Reconstr Surg 140, 116–124. 28654597 10.1097/PRS.0000000000003419

